# Acute Nonatherosclerotic Coronary Thromboembolism Presenting with an Inferior STEMI in a Patient on Oral Contraception

**DOI:** 10.1155/2021/5450376

**Published:** 2021-12-24

**Authors:** Nabil Braiteh, Raheel Chaudhry, Ibraheem Rehman, Jowana Breiteh, Alon Yarkoni

**Affiliations:** ^1^Department of Cardiology, United Health Services Hospitals, Wilson Regional Medical Center, NY, USA; ^2^Department of Internal Medicine, United Health Services Hospitals, Wilson Regional Medical Center, NY, USA; ^3^Emory University School of Medicine, Atlanta, Georgia, USA; ^4^Department of Nutrition and Food Sciences, American University of Beirut, Beirut, Lebanon

## Abstract

**Background:**

Direct coronary embolism in the setting of oral contraceptive pill (OCP) use is a rare adverse effect. It is known for OCP to increase the risk of thrombosis; however, leading to an inferior ST elevated myocardial infarction (STEMI) due to an acute occlusive embolism is a rare entity. Coronary embolism occurs in about 3% of patients with acute coronary syndrome. *Case Report*. We present a case of a young 41-year-old female with a past medical history significant for dysfunctional uterine bleeding on oral contraceptive pills, who presented to the hospital with chest pain. Her workup was significant for troponin elevation and an electrocardiogram showing inferior ST elevations. The patient was taken emergently to the cardiac catheterization lab. A coronary angiogram revealed a coronary thrombus involving the distal left main and proximal left anterior descending (LAD) with no evidence of atherosclerotic disease. The patient subsequently received anticoagulation therapy leading to complete resolution of symptoms and ST elevations.

**Conclusion:**

Coronary embolism is rare and often not considered in the differential of acute coronary syndrome. It is of utmost importance for clinicians to keep a wide differential of nonatherosclerotic causes of STEMI especially when the patient is young, without significant cardiac risk factors.

## 1. Introduction

Nonatherosclerotic causes of STEMI may be life-threatening and can occur even in the absence of significant cardiovascular risk factors. A nonatherosclerotic direct coronary thromboembolism is a rare occurrence. It requires further investigation and the initiation of appropriate therapy immediately.

Coronary embolism occurs in about 3% of patients with acute coronary syndrome [1]. It may be paradoxical, direct, or iatrogenic. An embolism breaks off an initial source such as a thrombus and then causes a decrease or blockage of blood flow upstream.

Oral contraceptive pills (OCPs) are used widely to prevent unwanted pregnancies and dysfunctional uterine bleeding. There has been much research on the increased risk of thromboembolic events from these drugs. Norgestimate/ethinyl estradiol is a progesterone and estrogen combination used to prevent ovulation and pregnancy. OCPs including norgestimate/ethinyl estradiol are known to significantly increase the risk of clot formation in female smokers who are above the age of 35 [2]. Estrogen is also known to cause downregulation of protein S creating a hypercoagulable state [3].

Herein we report a 41-year-old female who presented with an inferior STEMI and was found to have a saddle clot (thrombus) involving the distal left main artery, left circumflex, and proximal LAD. Her only cardiac risk factors were smoking and the use of oral contraceptives.

## 2. Case Report

A 41-year-old gravida 6 para 3 female (uncompleted pregnancies due to miscarriages) presented to the emergency department with a chief complaint of chest pain. Pain started two hours prior to presentation, was dull, left sided, substernal, radiated to her left arm, and with a severity of 7/10. One month prior to presentation the patient was complaining of an abnormal uterine bleeding and was started on oral norgestimate/ethinyl estradiol pills.

The patient has no significant past medical history. Her social history is significant for smoking 1/2 a pack per day for the past 10 years. Her family history is not significant for premature coronary artery disease or thrombosis. Her past surgical history is significant for dilatation and curettage two weeks prior to presentation. Home medications include daily oral norgestimate/ethinyl estradiol pills. Her only cardiac risk factors were smoking and the use of oral contraceptives.

**Figure 1:**
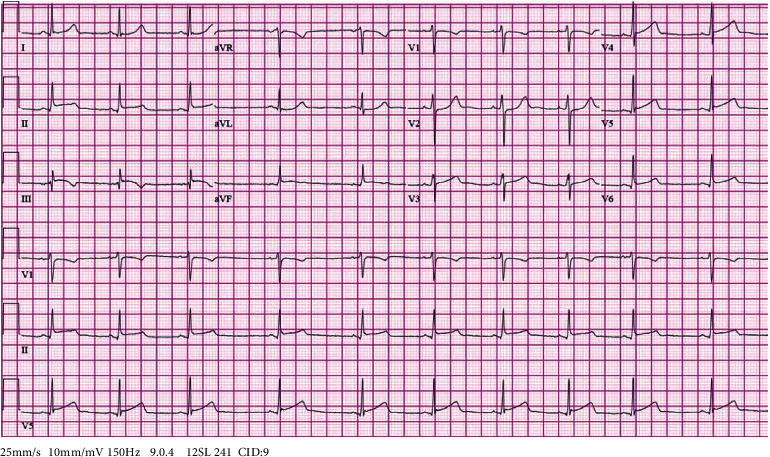
Electrocardiogram showing 1 mm ST elevations in inferior leads with no reciprocal changes and a sinus rhythm at a rate of 60 beats/minute.

Upon arrival to the emergency department, the patient was vitally stable. Her physical exam was noncontributory. Cardiac and lung exams were unremarkable. A twelve-lead ECG showed 1 mm ST elevations in inferior leads with no reciprocal changes and a sinus rhythm at a rate of 60 beats/minute **(**Figure 1**)**. A STEMI code was called, and the patient was taken emergently to the Cath Lab. She was started on acute coronary syndrome (ACS) treatment including oral aspirin 324 milligrams, oral clopidogrel 600 milligrams, and intravenous heparin 5000 U. Significant laboratory data showed a troponin of 0.615 ng/mL (0-0.04 ng/mL), hemoglobin of 8.7 g/dL (12.0-15.5 g/dL), and white blood cell count of 13.7 × 10^9^/L (4.5 − 11.0 × 10^9^/L).

**Figure 2:**
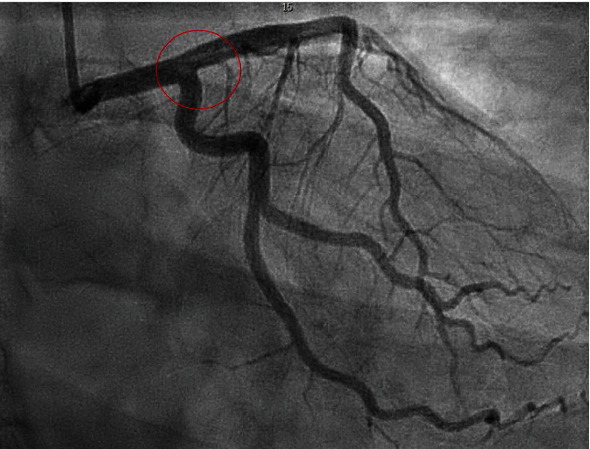
Coronary angiogram revealed a saddle clot (thrombus) involving the distal left main artery, left circumflex artery, and proximal left anterior descending artery (red circle).

Coronary angiogram revealed a saddle clot (thrombus) involving the distal left main artery, left circumflex, and proximal LAD **(**Figure 2**)**. TIMI flow grade was 3 throughout, and no intervention took place during the coronary angiogram. There was also an occlusion in the apical portion of the LAD. The right coronary artery did not have any abnormalities. An echocardiogram revealed an estimated ejection fraction of 55-60% with apical septal hypokinesis and a normal diastolic function. There was no evidence of an intra-atrial shunt or left atrial appendage. Patient was admitted to the cardiac care unit (CCU) and was started on intravenous tirofiban for 10 hours, oral aspirin 81 milligrams daily, oral clopidogrel 75 milligrams daily, and intravenous heparin drip as per ACS protocol. Troponin peaked at 11 ng/mL. The patient was given 2 units of PRBC due to persistent vaginal bleeding. Doppler of the lower extremities did not reveal any evidence of a DVT. Telemetry over 48 hours of the hospital stay did not show any evidence of cardiac arrhythmia.

Thrombophilia testing lab results showed antithrombin activity of 88% (80-120%), antithrombin III AG 79%, cardiolipin AB IGA < 9.4 APL (<20.0 APL), beta − 2 glycoprotein IGG < 9.4 U/mL (<20.0 U/mL), beta − 2 glycoprotein IGM < 9.4 U/mL (<20.0 U/mL), cardiolipin AB IGM MCLIP < 9.4 MPL (<15 MPL), cardiolipin AB IGG GCLIP < 9.4 GPL (<15 GPL), prothrombin G20210A gene negative, beta − 2 glycoprotein AB IGA < 9.4 U/mL (<15.0 U/mL), PS/PT IGG < 9.4 U (<30.0 U), PS/PT IGM 20.0 U (<30.0 U), and platelet count of 376 K/*μ*L (149-400 K/*μ*L).

**Figure 3:**
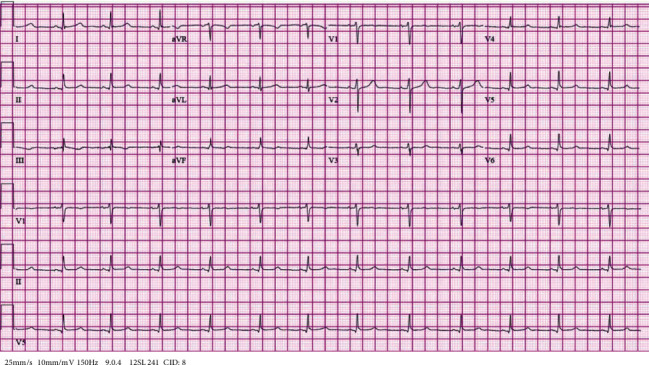
Electrocardiogram showing evidence of ST elevation resolution in the inferior leads.

OBGYN were consulted, and placement of IUD was recommended to help prevent bleeding. After 48 hours, heparin was discontinued. EKG prior to discharge showed resolution of ST elevations (Figure 3). A repeat angiography was not done as symptoms had resolved and EKG showed resolution of STEMI. OCPs were held, and patient was discharged on oral anticoagulation.

## 3. Discussion

Coronary embolism occurs in about 3% of patients with acute coronary syndrome. However, it is a rare occurrence in a patient without a cardiac history of endocarditis, arrhythmia, or family history. Coronary embolism is of three main types: direct, paradoxical, and iatrogenic.

Direct embolism usually results from a thrombus originating in the left ventricle or left atrium or due to endocarditis involving the mitral or aortic valve. Paradoxical embolism occurs from the venous circulation through a patent foramen ovale. Iatrogenic embolism occurs after a cardiac procedure [1].

Risk factors include evidence of blood stasis (i.e., left ventricular aneurysm, atrial fibrillation, and deep vein thrombosis with ASD or PFO), hypercoagulable state (i.e., cancer, thrombophilia, oral contraceptive use, and heparin-induced thrombocytopenia), endothelial injury (i.e., angioplasty, valvuloplasty, and aortic/coronary surgery), and anatomic predisposition (i.e., ASD/PFO, endocarditis, and mitral stenosis). Smoking itself is considered to increase the risk of ischemic vascular events such as myocardial infarction and ischemic strokes [4–6].

Diagnosis of coronary embolism can be made using a scoring system proposed by Shibata et al. [7]. Major criteria include (1) angiographic evidence of coronary embolus, (2) concomitant systemic embolization without evidence of left ventricular thrombus, (3) concomitant coronary emboli in multiple coronary territories, (4) evidence of an embolic source based on imaging, and (5) histological evidence of venous origin of coronary embolic material.

Minor criteria include (1) <25% stenosis on angiography in the nonculprit vessels, (2) presence of atrial fibrillation, and (3) presence of embolic risk factors.

Patients with 2 or more major criteria, 1 major and 2 minor, or 3 minor criteria were considered to have a definite coronary embolus. Patients with 1 major and 1 minor or 2 minor criteria were considered to have a probable coronary embolus [1, 7].

Our patient is a young female with tobacco abuse, on norgestimate/ethinyl estradiol pills for dysfunctional uterine bleeding who presented with an inferior STEMI secondary to a clot (thrombus) involving the distal left main artery, left circumflex, and proximal and distal LAD.

ECG changes were mainly seen in the inferior rather than the anterior or anterolateral leads. That can be explained by two theories. (1) There might have been a right coronary artery clot that was dislodged prior to the angiogram especially that the repeat ECG showed resolution of inferior ST elevations. (2) There was a complete occlusion of the distal LAD artery (which is a wrap-around artery), with evidence of continued flow in the left main and proximal LAD artery.

She did not have a history of atrial fibrillation; no evidence of clot, valvular heart disease, wall motion abnormality, or an intra-atrial shunt with rest and provocation on TTE; and no evidence of DVT. The primary hypothesis for our case is that she had a direct arterial thromboembolism that developed secondary to the combination of oral contraception and smoking which is a rare entity, since most thromboembolisms due to acquired thrombophilia are rather venous. Although the patient was monitored on telemetry over 48 hours without cardiac arrhythmias, she was recommended to have a long-term cardiac monitoring (by an event monitor) to rule out paroxysmal atrial fibrillation as a possible cause of this thromboembolism. A transesophageal echocardiogram was recommended to be done in the outpatient setting to further assess the presence of a clot in the left atrial appendage.

Although thrombophilia workup is seldom indicated as part of the coronary embolism workup [1], it was performed during her admission and was negative. The patient did have miscarriages in the past, but it is less likely due to a hypercoagulable state since that workup was negative in the past. Long-term cohort studies revealed increased risk of venous rather than arterial thromboembolism in patients with inherited thrombophilias [8].

Antiplatelet therapy was continued in our case due to the presence of a distal LAD (apical) occlusion which is most likely due to the presence of a clot (thrombus), but we were unable to completely rule out the presence of atherosclerotic disease in that area.

Although estrogen and progesterone therapy may be an effective method of dysfunctional uterine bleeding, it is an oral contraceptive which is known to create a hypercoagulable state in combination with smoking, thereby increasing the risk for a thromboembolic event [9–11].

Vendittelli et al. performed a literature review on a total of 214 cases of coronary thromboembolism with the etiology being atrial fibrillation (26%), endocarditis (24%), iatrogenic emboli (21%), prosthetic valve thrombi (12%), hypercoagulable state with PFO (6%), aortic atheroma (5%), myxoma (2%), fat emboli (2%), and coronary stent emboli (2%) [12].

Treatment of coronary embolism depends on the root cause. Patients with atrial fibrillation or recurrent coronary embolism should be offered long-term anticoagulation regardless of their CHADS2-VASc score. Patients with a reversible risk factor such as smoking or OCP use should receive oral anticoagulation for 3 months [1].

After review of the literature, there have been only a couple of case reports to describe a case of a STEMI due to coronary embolism in a patient who is taking OCPs without evidence of a DVT or a patent foramen ovale [13, 14].

## 4. Conclusion

Atherosclerotic coronary artery disease is the main cause of STEMI. While coronary embolism is rare and often not considered in the differential of acute coronary syndrome, it is of utmost importance for clinicians to keep a wide differential of nonatherosclerotic causes of STEMI especially when the patient is young, without significant cardiac risk factors.
